# Supplementary material for: Distinct mechanisms for TMPRSS2 expression explain organ-specific inhibition of SARS-CoV-2 infection by enzalutamide
Source: Nat Commun. 2021 Feb 8;12:866. doi: 10.1038/s41467-021-21171-x (PMC7870838; doi:10.1038/s41467-021-21171-x)
Supplement: Supplementary file 1 — Supplementary Information [file 41467_2021_21171_MOESM1_ESM.pdf]

Supplementary Information

Distinct mechanisms for TMPRSS2 expression explain organ-specific inhibition of SARS-CoV-2 infection by enzalutamide

Fei Li, Ming Han, Pengfei Dai, Wei Xu, Juan He, Xiaoting Tao, Yang Wu, Xinyuan Tong, Xinyi Xia, Wangxin Guo, Yunjiao Zhou, Yunguang Li, Yiqin Zhu, Xiaoyu Zhang, Zhuang Liu, Rebiguli Aji, Xia Cai, Yutang Li, Di Qu, Yu Chen, Shibo Jiang, Qiao Wang, Hongbin Ji, Youhua Xie, Yihua Sun, Lu Lu & Dong Gao

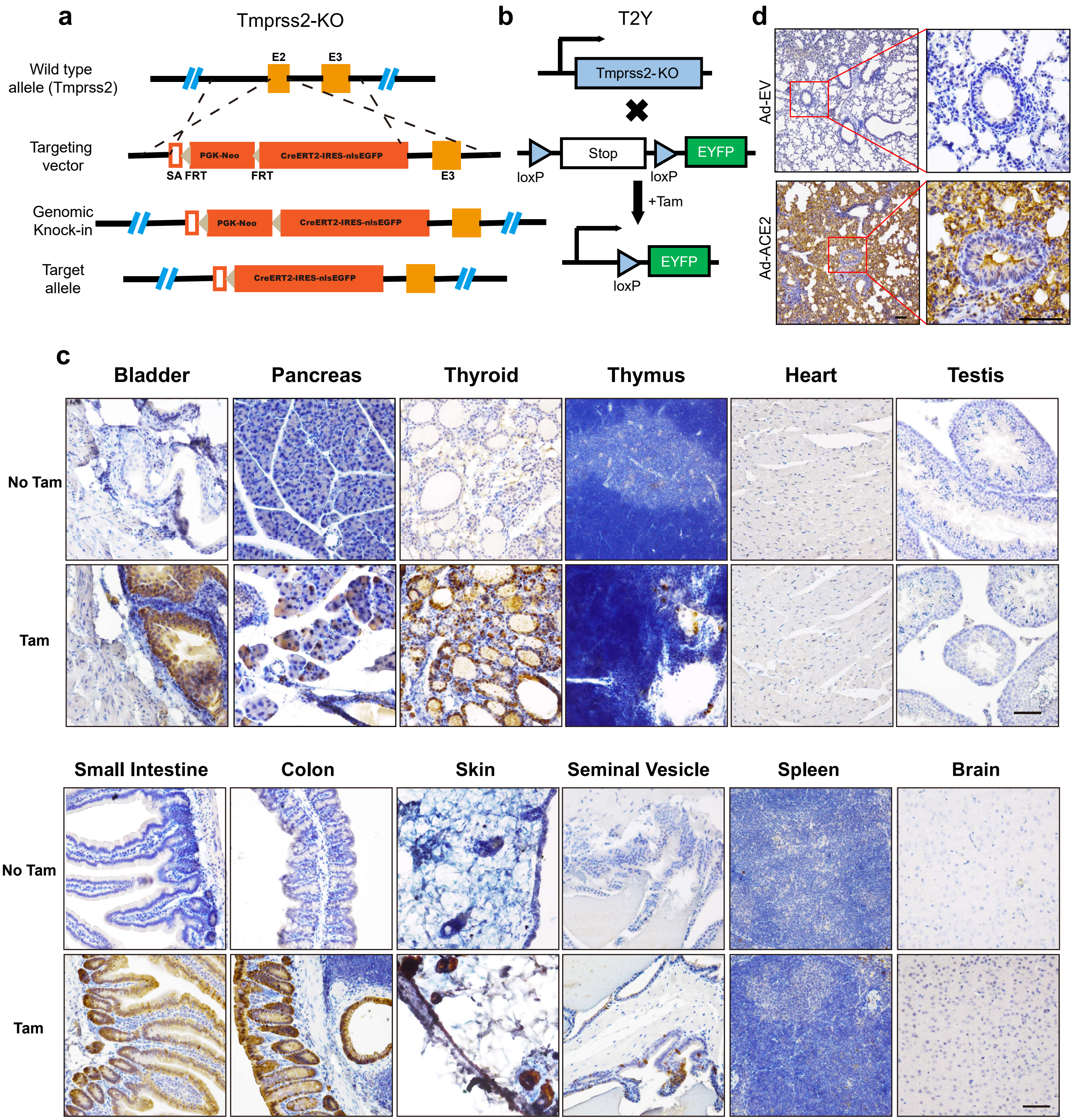
a
Tmprss2-KO
Wild type allele (Tmprss2)
E2
E3
Targeting vector
PGK-Neo
CreERT2-IRES-nlsEGFP
SA FRT
FRT
E3
Genomic Knock-in
PGK-Neo
CreERT2-IRES-nlsEGFP
Target allele
CreERT2-IRES-nlsEGFP
b
T2Y
Tmprss2-KO
Stop
EYFP
loxP
loxP
+Tam
EYFP
loxP
d
Ad-EV
Ad-ACE2
c
Bladder
Pancreas
Thyroid
Thymus
Heart
Testis
No Tam
Tam
Small Intestine
Colon
Skin
Seminal Vesicle
Spleen
Brain
No Tam
Tam

Supplementary Figure 1 Identification of Tmprss2-positive cells in multiple mouse organs. (a) Construction strategy for Tmprss2-KO mice. **(b)** Breeding strategy for the generation of T2Y mice. **(c)** YFP IHC staining for multiple organs of T2Y mice with (bottom) or without (top) tamoxifen gavage. **(d)** FLAG IHC staining in the lungs of WT transduced with Ad-EV (top) or Ad-ACE2 transduction (bottom), samples were collected 5 days post transduction. Scale bars represent 50 μm.

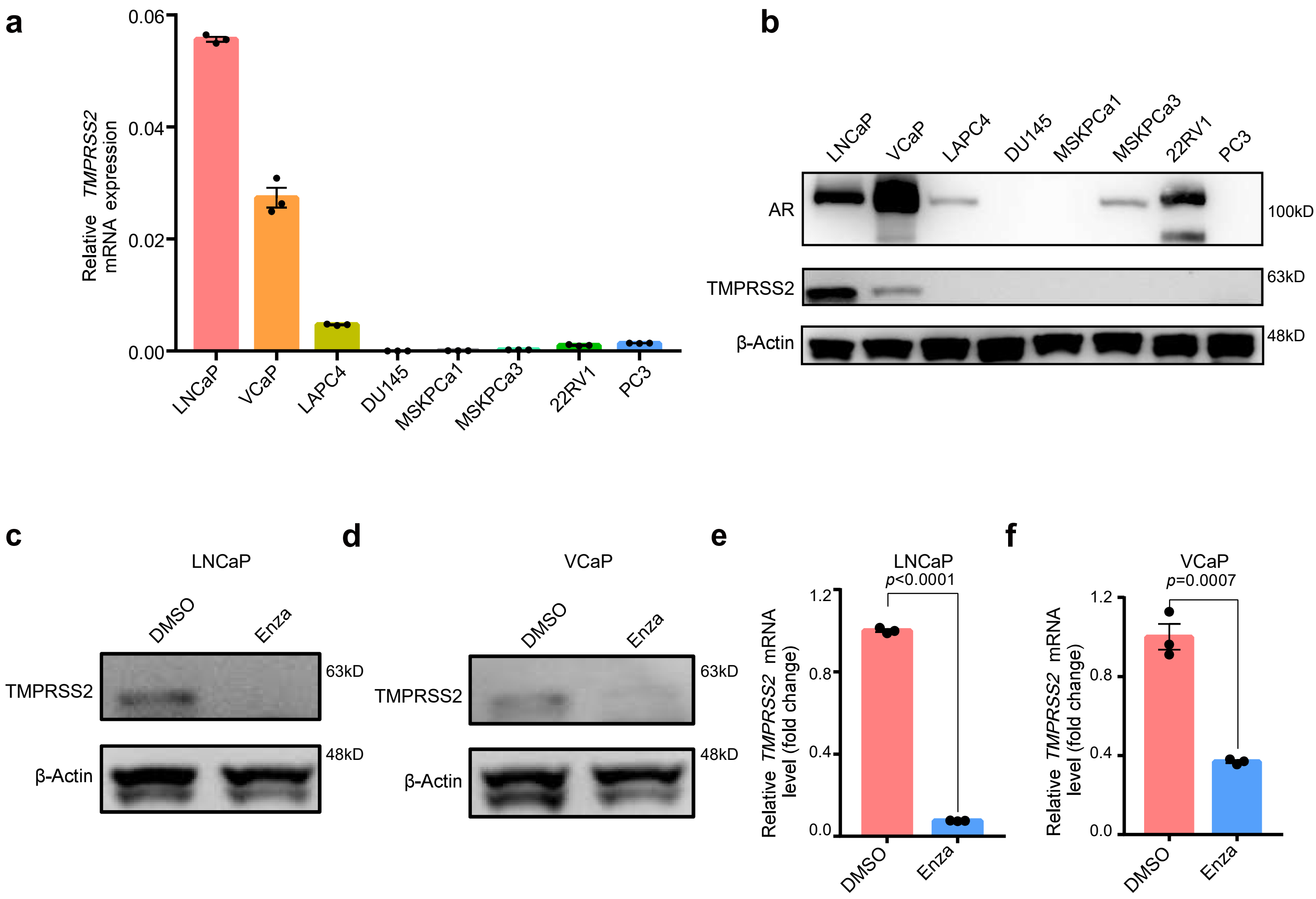
a
Relative TMPRSS2 mRNA expression
0.06
0.04
0.02
0.00
LNCaP
VCaP
LAPC4
DU145
MSKPCa1
MSKPCa3
22RV1
PC3
b
LNCaP
VCaP
LAPC4
DU145
MSKPCa1
MSKPCa3
22RV1
PC3
AR
100kD
TMPRSS2
63kD
β-Actin
48kD
c
LNCaP
DMSO
Enza
TMPRSS2
63kD
β-Actin
48kD
d
VCaP
DMSO
Enza
TMPRSS2
63kD
β-Actin
48kD
e
LNCaP
p<0.0001
Relative TMPRSS2 mRNA level (fold change)
1.2
0.8
0.4
0.0
DMSO
Enza
f
VCaP
p=0.0007
Relative TMPRSS2 mRNA level (fold change)
1.2
0.8
0.4
0.0
DMSO
Enza

Supplementary Figure 2 TMPRSS2 expression is reduced by enzalutamide in LNCaP and VCaP cells. (a) qRT-PCR analysis of *TMPRSS2* mRNA expression in multiple prostate cancer cell lines (LNCaP, VCaP, LAPC4, DU145, 22RV1 and PC3) and two prostate cancer organoid lines (MSKPCa1 and MSKPCa3) (mean ± SEM, n=3 biologically independent samples). **(b)** Western blotting analysis of TMPRSS2 and AR expression in multiple prostate cancer cell lines and two prostate cancer organoid lines. Source data are provided as a Source Data file. **(c)** Western blotting analysis of TMPRSS2 expression in LNCaP cells treated with DMSO under normal FBS condition or enzalutamide treatment under charcoal stripped FBS condition. Source data are provided as a Source Data file. **(d)** Western blotting analysis of TMPRSS2 expression in VCaP cells treated with DMSO under normal FBS condition or enzalutamide treatment under charcoal stripped FBS condition. Source data are provided as a Source Data file. **(e)** qRT-PCR analysis of *TMPRSS2* mRNA expression in LNCaP cells treated with DMSO under normal FBS condition or enzalutamide treatment under charcoal stripped FBS condition (two-tailed t-test, mean ± SEM, n=3 biologically independent experiments). **(f)** qRT-PCR analysis of *TMPRSS2* mRNA expression in VCaP cells treated with DMSO under normal FBS condition or enzalutamide treatment under charcoal stripped FBS condition (two-tailed t-test, mean ± SEM, n=3 biologically independent experiments).

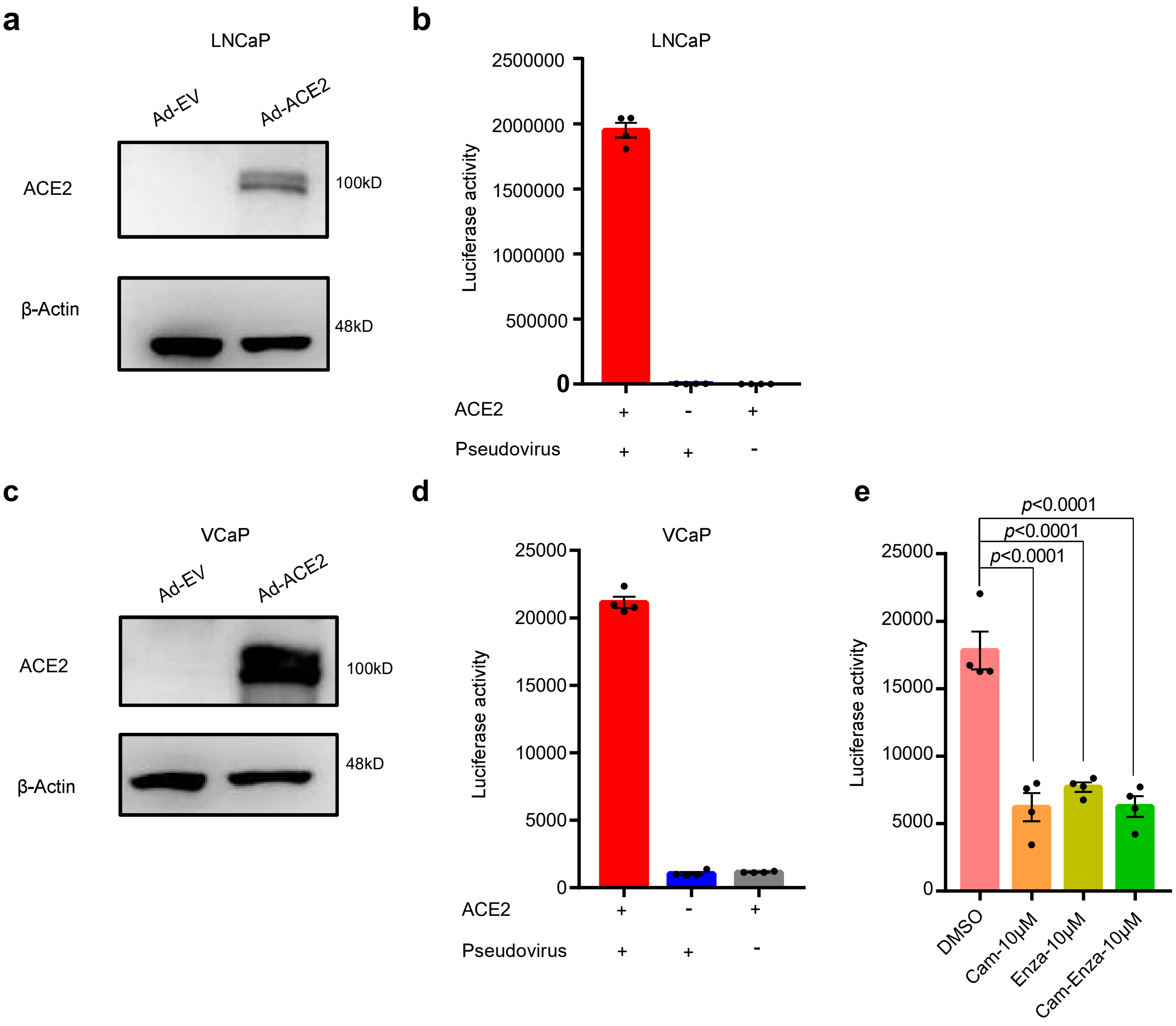

a
LNCaP
Ad-EV
Ad-ACE2
ACE2
100kD
β-Actin
48kD
b
LNCaP
Luciferase activity
2500000
2000000
1500000
1000000
500000
0
ACE2
+
-
+
Pseudovirus
+
+
-
c
VCaP
Ad-EV
Ad-ACE2
ACE2
100kD
β-Actin
48kD
d
VCaP
Luciferase activity
25000
20000
15000
10000
5000
0
ACE2
+
-
+
Pseudovirus
+
+
-
e
p<0.0001
p<0.0001
p<0.0001
Luciferase activity
25000
20000
15000
10000
5000
0
DMSO
Cam-10μM
Enza-10μM
Cam-Enza-10μM

Supplementary Figure 3 Enzalutamide significantly reduces infection of VCaP cell with SARS-CoV-2-S. (a) Western blotting analysis of ACE2 and β-Actin expression in LNCaP cells with empty vector adenovirus transduction or ACE2 adenovirus transduction. Source data are provided as a Source Data file. **(b)** SARS-CoV-2-S-driven entry into LNCaP cells with or without Ad-ACE2 transduction. Luciferase activity was measured 48 hours post SARS-CoV-2-S infection (mean ± SEM, n=4 biologically independent samples). **(c)** Western blotting analysis of ACE2 and β-Actin expression in VCaP cells with empty vector adenovirus transduction or ACE2 adenovirus transduction. Source data are provided as a Source Data file. **(d)** SARS-CoV-2-S-driven entry into VCaP cells with or without Ad-ACE2 transduction, luciferase activity was measured 48 hours post SARS-CoV-2-S infection (mean ± SEM, n=4 biologically independent samples). **(e)** SARS-CoV-2-S-driven entry into VCaP cells with Ad-ACE2 transduction treated with DMSO, 10 μM camostat mesylate, 10 μM enzalutamide and 10 μM camostat mesylate/enzalutamide. Luciferase activity was measured 48 hours post SARS-CoV-2-S infection (one-way ANOVA and Tukey's test, mean ± SEM, n=4 biologically independent samples).

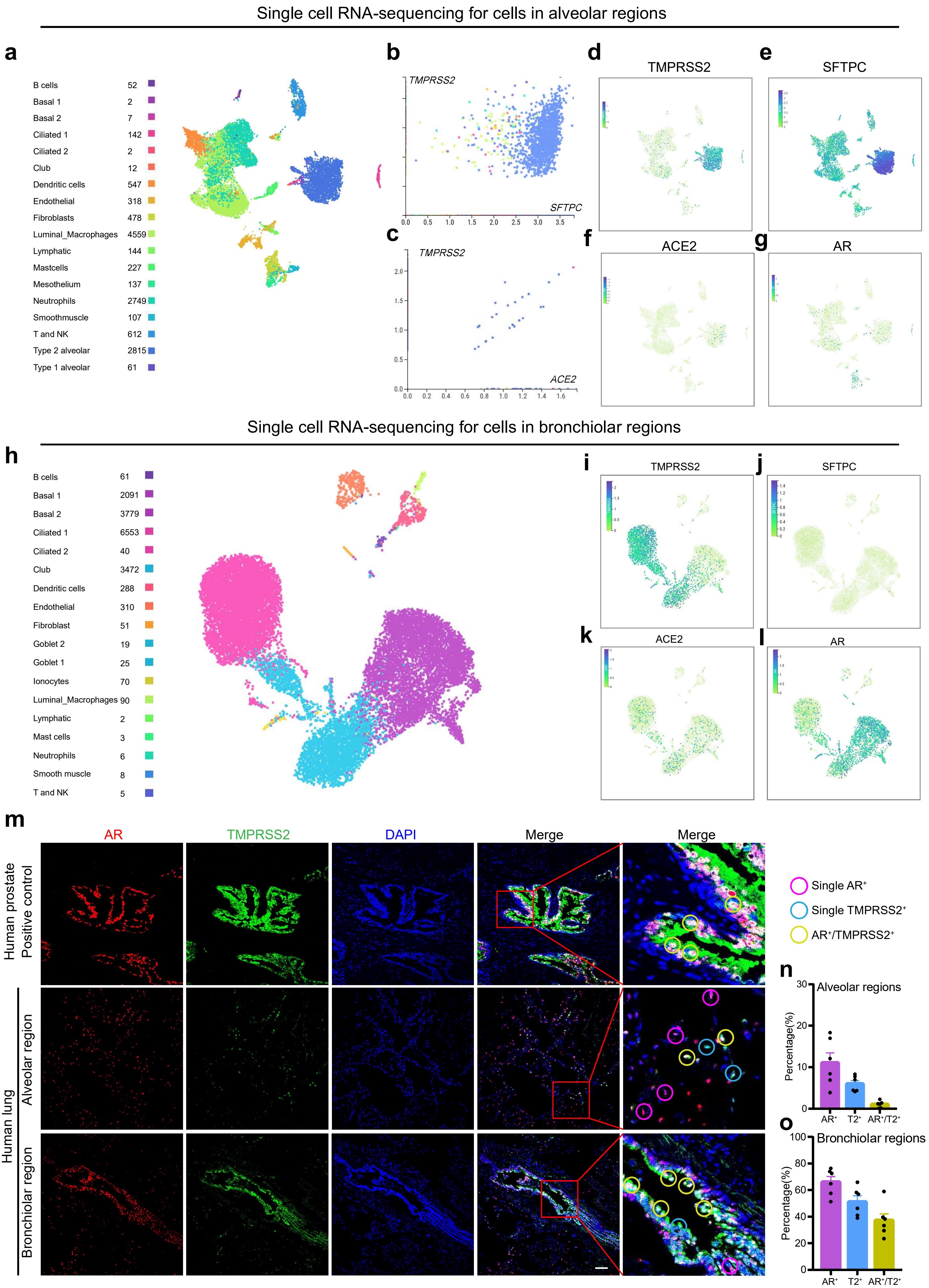
Single cell RNA-sequencing for cells in alveolar regions
a
B cells 52
Basal 1 2
Basal 2 7
Ciliated 1 142
Ciliated 2 2
Club 12
Dendritic cells 547
Endothelial 318
Fibroblasts 478
Luminal_Macrophages 4559
Lymphatic 144
Mastcells 227
Mesothelium 137
Neutrophils 2749
Smoothmuscle 107
T and NK 612
Type 2 alveolar 2815
Type 1 alveolar 61
b
TMPRSS2
SFTPC
c
TMPRSS2
ACE2
d
TMPRSS2
e
SFTPC
f
ACE2
g
AR
Single cell RNA-sequencing for cells in bronchiolar regions
h
B cells 61
Basal 1 2091
Basal 2 3779
Ciliated 1 6553
Ciliated 2 40
Club 3472
Dendritic cells 288
Endothelial 310
Fibroblast 51
Goblet 2 19
Goblet 1 25
Ionocytes 70
Luminal_Macrophages 90
Lymphatic 2
Mast cells 3
Neutrophils 6
Smooth muscle 8
T and NK 5
i
TMPRSS2
j
SFTPC
k
ACE2
l
AR
m
AR
TMPRSS2
DAPI
Merge
Merge
Human prostate Positive control
Human lung
Alveolar region
Bronchiolar region
Single AR+
Single TMPRSS2+
AR+/TMPRSS2+
n
Alveolar regions
Percentage(%)
AR+
T2+
AR+/T2+
o
Bronchiolar regions
Percentage(%)
AR+
T2+
AR+/T2+

Supplementary Figure 4 AR/TMPRSS2 double positive cells are verified in human lungs. (a) UMAP plot displaying cell clusters of cells from human lung alveolar regions in a public dataset using COVID-19 Cell Atlas. **(b)** *TMPRSS2* and *SFTPC* mRNA expression across multiple cell types. **(c)** *TMPRSS2* and *ACE2* mRNA expression across multiple cell types. **(d-g)** *TMPRSS2* **(d)**, *SFTPC* **(e)**, *ACE2* **(f)** and *AR* **(g)** mRNA expression levels on UMAP plots across multiple cell types in **(a)**. **(h)** UMAP plot displaying cell clusters of cells from human lung bronchiolar regions in a public dataset using COVID-19 Cell Atlas. **(i-l)** *TMPRSS2* **(i)**, *SFTPC* **(j)**, *ACE2* **(k)** and *AR* **(l)** mRNA expression levels on UMAP plots across multiple cell types in **(h)**. **(m)** Immunofluorescence staining of AR and TMPRSS2 in human prostates (top) and lungs (middle and bottom). **(n)** Quantification for AR-positive, TMPRSS2-positive and AR/TMPRSS2 double positive cells in human lung alveolar regions (mean ± SEM, n=6 biologically independent samples). **(o)** Quantification for AR-positive, TMPRSS2-positive and AR/TMPRSS2 double positive cells in human lung bronchiolar regions (mean ± SEM, n=6 biologically independent samples). Scale bars represent 50 μm.

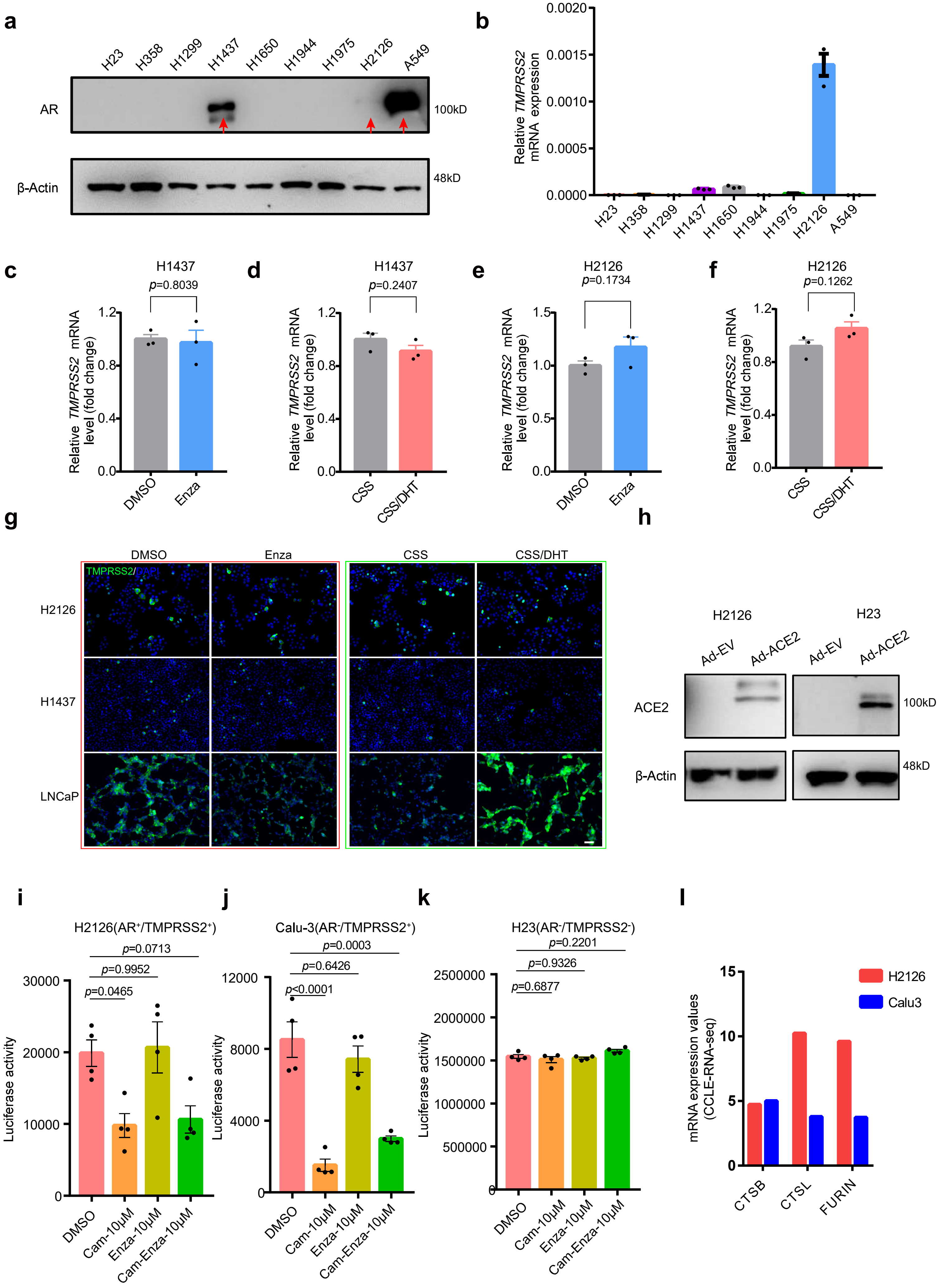
a
H23
H358
H1299
H1437
H1650
H1944
H1975
H2126
A549
AR
100kD
β-Actin
48kD
b
Relative TMPRSS2 mRNA expression
0.0020
0.0015
0.0010
0.0005
0.0000
H23
H358
H1299
H1437
H1650
H1944
H1975
H2126
A549
c
H1437
p=0.8039
Relative TMPRSS2 mRNA level (fold change)
DMSO
Enza
d
H1437
p=0.2407
CSS
CSS/DHT
e
H2126
p=0.1734
DMSO
Enza
f
H2126
p=0.1262
CSS
CSS/DHT
g
DMSO
Enza
CSS
CSS/DHT
TMPRSS2/DAPI
H2126
H1437
LNCaP
h
H2126
H23
Ad-EV
Ad-ACE2
Ad-EV
Ad-ACE2
ACE2
100kD
β-Actin
48kD
i
H2126(AR+/TMPRSS2+)
p=0.0713
p=0.9952
p=0.0465
Luciferase activity
DMSO
Cam-10μM
Enza-10μM
Cam-Enza-10μM
j
Calu-3(AR-/TMPRSS2+)
p=0.0003
p=0.6426
p<0.0001
Luciferase activity
DMSO
Cam-10μM
Enza-10μM
Cam-Enza-10μM
k
H23(AR-/TMPRSS2-)
p=0.2201
p=0.9326
p=0.6877
Luciferase activity
DMSO
Cam-10μM
Enza-10μM
Cam-Enza-10μM
l
mRNA expression values (CCLE-RNA-seq)
H2126
Calu3
CTSB
CTSL
FURIN

Supplementary Figure 5 Enzalutamide does not prevent SARS-CoV-2-S-driven entry into lung cells. **(a)** Western blotting analysis of AR expression in multiple lung cancer cells. Source data are provided as a Source Data file. **(b)** qRT-PCR analysis of *TMPRSS2* mRNA expression in multiple lung cancer cells (mean ± SEM, n=3 biologically independent samples). **(c)** qRT-PCR analysis of *TMPRSS2* mRNA expression in H1437 cells with vehicle or enzalutamide treatment (two-tailed t-test, mean ± SEM, n=3 biologically independent samples). **(d)** qRT-PCR analysis of *TMPRSS2* mRNA expression in H1437 cells with or without DHT treatment under charcoal stripped FBS condition (two-tailed t-test, mean ± SEM, n=3 biologically independent samples). **(e)** qRT-PCR analysis of *TMPRSS2* mRNA expression in H2126 cells with vehicle or enzalutamide treatment (two-tailed t-test, mean ± SEM, n=3 biologically independent samples). **(f)** qRT-PCR analysis of *TMPRSS2* mRNA expression in H2126 cells with or without DHT treatment under charcoal stripped FBS condition (two-tailed t-test, mean ± SEM, n=3 biologically independent samples). **(g)** Immunofluorescence staining of TMPRSS2 in H2126 (top), H1437 (middle) and LNCaP (bottom) cells under multiple treatments condition. **(h)** Western blotting analysis of ACE2 and β-Actin expression in H2126 (left) and H23 (right) cells with empty vector adenovirus transduction or ACE2 adenovirus transduction respectively. Source data are provided as a Source Data file. **(i-k)** SARS-CoV-2-S-driven entry into H2126 (**i**), Calu-3 (**j**) and H23 (**k**) cells transduced with Ad-ACE2 and treated with DMSO, 10 μM camostat mesylate, 10 μM enzalutamide and 10 μM camostat mesylate/enzalutamide. Luciferase activity was measured 48 hours post SARS-CoV-2-S infection (one-way ANOVA and Tukey's test, mean ± SEM, n=4 biologically independent samples). **(l)** CCLE mRNA expression levels of *CTSB*, *CTSL and FURIN* in H2126 and Calu3 cells. Scale bars represent 50 μm.

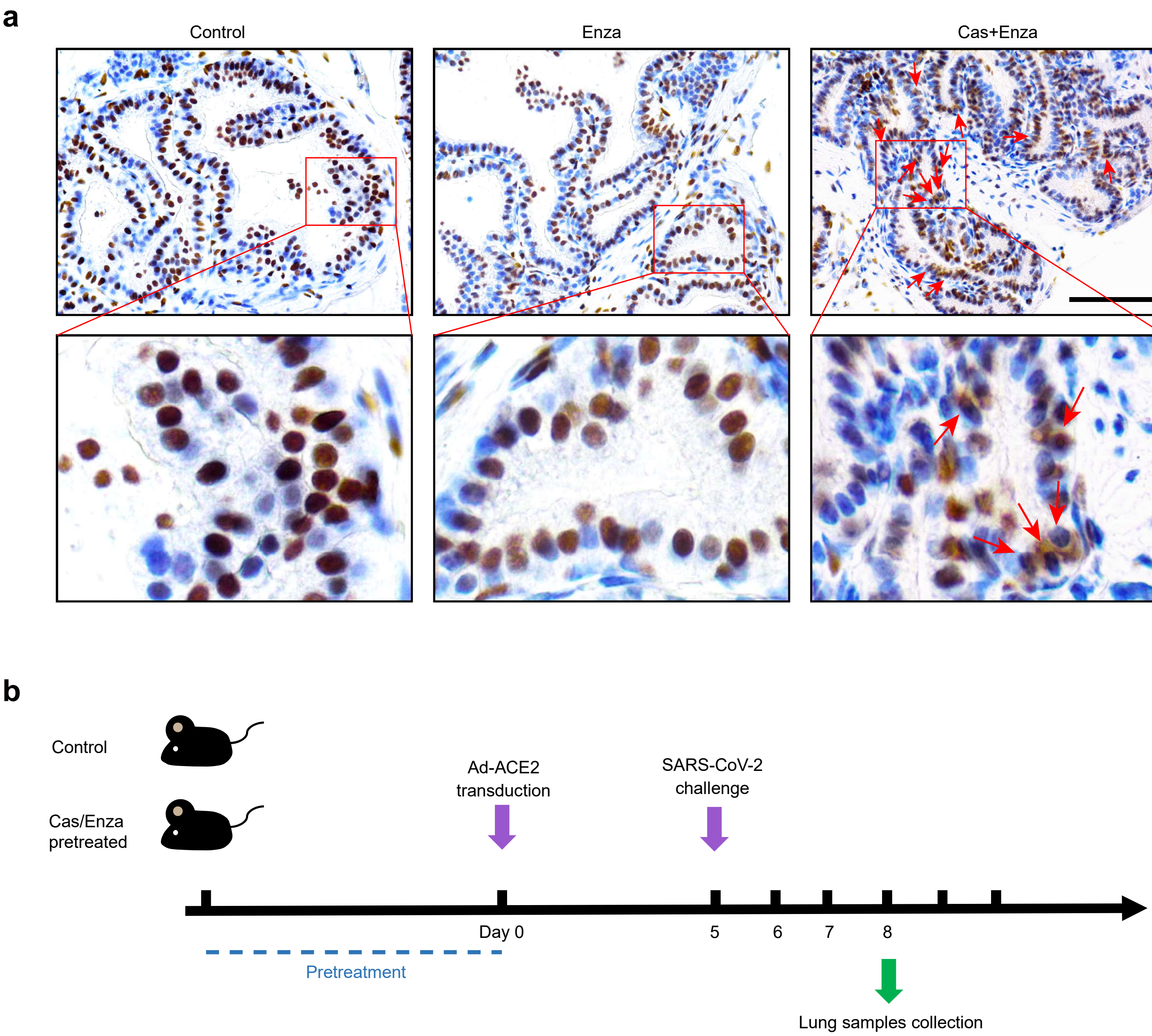
a
Control
Enza
Cas+Enza
b
Control
Cas/Enza
pretreated
Ad-ACE2
transduction
SARS-CoV-2
challenge
Day 0
5
6
7
8
Pretreatment
Lung samples collection

Supplementary Figure 6 Ad-ACE2-transduced mouse models are employed to assess the efficacy of enzalutamide *in vivo*. (a) IHC staining for AR in the prostates of wild type mice, enzalutamide-treated mice and enzalutamide-treated castrated mice respectively. Red arrows indicate blocking of nuclear translocation of AR in the prostates of enzalutamide treated castrated mice. **(b)** Schematic strategy for Ad-ACE2-transduced mouse to evaluate the therapeutic efficacy of enzalutamide. Scale bars represent 50 μm.

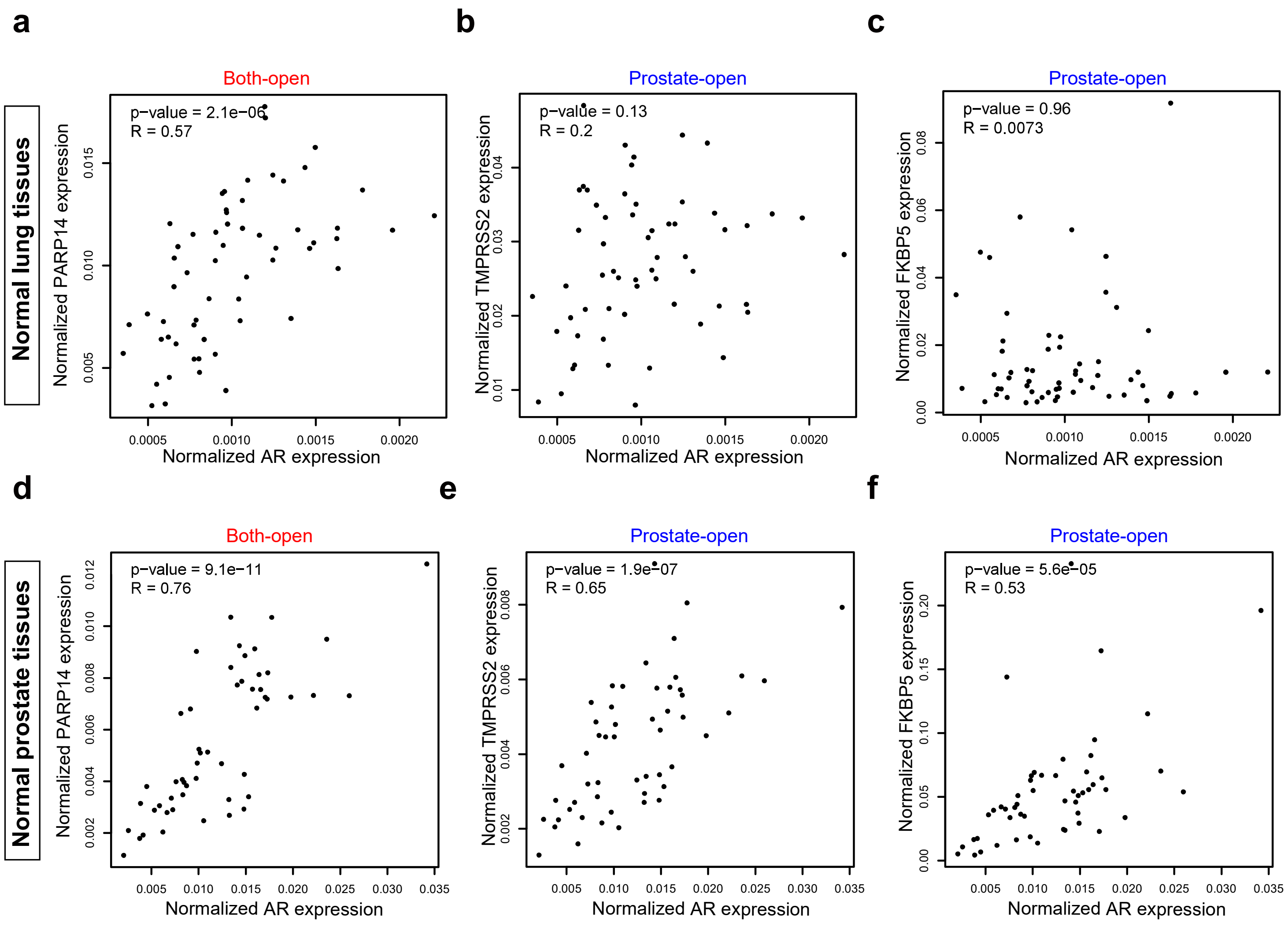
a
b
c
Normal lung tissues
Both-open
p−value = 2.1e−06
R = 0.57
Normalized PARP14 expression
Normalized AR expression
Prostate-open
p−value = 0.13
R = 0.2
Normalized TMPRSS2 expression
Normalized AR expression
Prostate-open
p−value = 0.96
R = 0.0073
Normalized FKBP5 expression
Normalized AR expression
d
e
f
Normal prostate tissues
Both-open
p−value = 9.1e−11
R = 0.76
Normalized PARP14 expression
Normalized AR expression
Prostate-open
p−value = 1.9e−07
R = 0.65
Normalized TMPRSS2 expression
Normalized AR expression
Prostate-open
p−value = 5.6e−05
R = 0.53
Normalized FKBP5 expression
Normalized AR expression

Supplementary Figure 7 Correlation analysis of mRNA levels of AR and TMPRSS2 in human lung tissues and prostate tissues. (a-c) Correlation analysis for normalized expression of *AR* and a both-open gene *PARP14* (left) **(a)**, two prostate-open genes *TMPRSS2* (middle) **(b)** and *FKBP5* (right) respectively in normal lung tissues using TCGA datasets **(c)** (Pearson correlation test). **(d-f)** Correlation analysis for normalized expression of *AR* and a both-open gene *PARP14* (left) **(d)**, two prostate-open genes *TMPRSS2* (middle) **(e)** and *FKBP5* (right) respectively in normal prostate tissues using TCGA datasets **(f)** (Pearson correlation test).

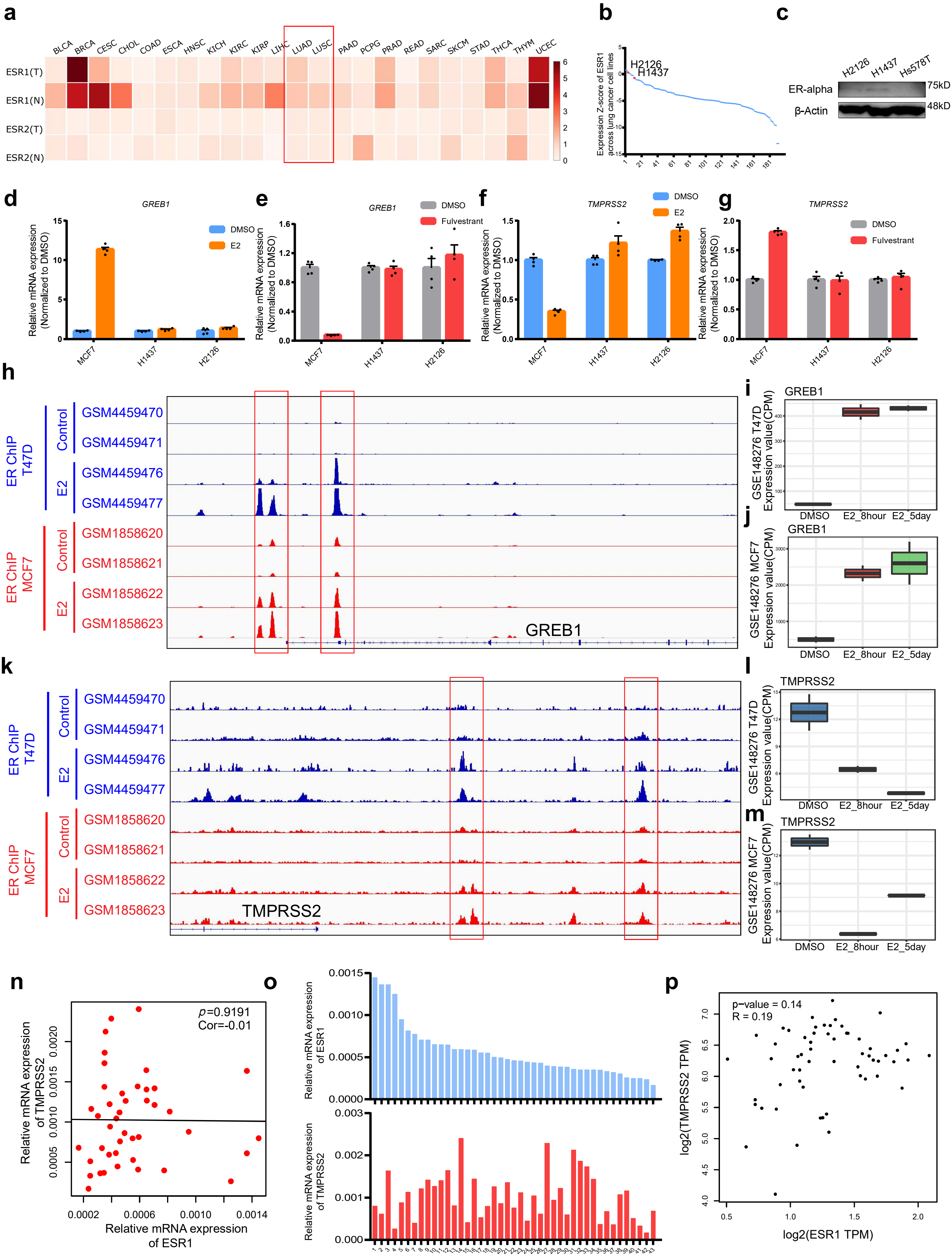

a
BLCA BRCA CESC CHOL COAD ESCA HNSC KICH KIRC KIRP LIHC LUAD LUSC PAAD PCPG PRAD READ SARC SKCM STAD THCA THYM UCEC
ESR1(T)
ESR1(N)
ESR2(T)
ESR2(N)
b
Expression Z-score of ESR1 across lung cancer cell lines
H2126
H1437
c
H2126 H1437 Hs578T
ER-alpha
β-Actin
75kD
48kD
d
GREB1
DMSO
E2
Relative mRNA expression (Normalized to DMSO)
MCF7 H1437 H2126
e
GREB1
DMSO
Fulvestrant
f
TMPRSS2
g
TMPRSS2
h
ER ChIP T47D
Control
E2
GSM4459470
GSM4459471
GSM4459476
GSM4459477
ER ChIP MCF7
GSM1858620
GSM1858621
GSM1858622
GSM1858623
GREB1
i
GREB1
GSE148276 T47D Expression value(CPM)
DMSO E2_8hour E2_5day
j
GREB1
GSE148276 MCF7 Expression value(CPM)
k
TMPRSS2
l
TMPRSS2
m
TMPRSS2
n
p=0.9191
Cor=-0.01
Relative mRNA expression of TMPRSS2
Relative mRNA expression of ESR1
o
Relative mRNA expression of ESR1
Relative mRNA expression of TMPRSS2
p
p−value = 0.14
R = 0.19
log2(TMPRSS2 TPM)
log2(ESR1 TPM)

Supplementary Figure 8 Organ-specific regulation of TMPRSS2 by ER. (a) Heatmap for mRNA expression levels of *ESR1* and *ESR2* across multiple tissue or organ types (T: Tumor tissue, N: Normal tissue) **(b)** mRNA Expression Z-score of *ESR1* across lung cancer cell lines. **(c)** Western blotting for ER-alpha and β-Actin in H2126, H1437 and Hs578T (negative control) cells. Source data are provided as a Source Data file. **(d)** qRT-PCR analysis of *GREB1* mRNA expression in MCF7, H1437 and H2126 cells 24 hours post DMSO or E2 treatment in medium containing charcoal-stripped serum (mean ± SEM, n=4 biologically independent samples). **(e)** qRT-PCR analysis of *GREB1* mRNA expression in MCF7, H1437 and H2126 cells 24 hours post DMSO or fulvestrant treatment in medium containing normal serum (mean ± SEM, n=4 biologically independent samples). **(f)** qRT-PCR analysis of *TMPRSS2* mRNA expression in MCF7, H1437 and H2126 cells 24 hours post DMSO or E2 treatment in medium containing charcoal-stripped serum (mean ± SEM, n=4 biologically independent samples). **(g)** qRT-PCR analysis of *TMPRSS2* mRNA expression in MCF7, H1437 and H2126 cells 24 hours post DMSO or fulvestrant treatment in medium containing normal serum (mean ± SEM, n=4 biologically independent samples). **(h)** IGV snapshot representing ER binding peaks in *GREB1* locus. **(i-j)** CPM value for mRNA expression of *GREB1* in T47D **(i)** and MCF7 cells **(j)** with or without E2 treatment (upper whisker=largest observation less than or equal to upper hinge+1.5*IQR, upper hinge=75% quantile, median, lower hinge=25% quantile and lower whisker= mallest observation greater than or equal to lower hinge-1.5*IQR, n=2 biologically independent samples). **(k)** IGV snapshot representing ER binding peaks in *TMPRSS2* locus in T47D and MCF7 cells with or without E2 treatment. **(l-m)** CPM value for mRNA expression of *TMPRSS2* in T47D **(l)** and MCF7 cells **(m)** with or without E2 treatment (upper whisker=largest observation less than or equal to upper hinge+1.5*IQR, upper hinge=75% quantile, median, lower hinge=25% quantile and lower whisker=smallest observation greater than or equal to lower hinge-1.5*IQR, n=2 biologically independent samples). **(n)** Correlation analysis for relative mRNA expression of *ESR1* and *TMPRSS2* in freshly dissociated human lung cells from 43 normal lung tissues using qRT-PCR (Pearson correlation test). **(o)** Relative mRNA expression of *ESR1* (top) and *TMPRSS2* (bottom) in freshly dissociated lung cells from 43 normal lung tissue samples. **(p)** Correlation analysis for normalized mRNA expression (TPM) of *ESR1* and *TMPRSS2* in normal lung tissues using TCGA datasets (Pearson correlation test).
