# Supplementary material for: Distinct mechanisms for TMPRSS2 expression explain organ-specific inhibition of SARS-CoV-2 infection by enzalutamide
Source: Nat Commun. 2021 Feb 8;12:866. doi: 10.1038/s41467-021-21171-x (PMC7870838; doi:10.1038/s41467-021-21171-x)
Supplement: Supplementary file 3 — Description of Additional Supplementary Files [file 41467_2021_21171_MOESM3_ESM.docx]

File Name: Supplementary Data 1
Description: Peak file for “both-open” sites.

File Name: Supplementary Data 2
Description: Peak file for “prostate-open” sites.

File Name: Supplementary Data 3
Description: List for all primers used in this study.
