# Supplementary material for: Distinct mechanisms for TMPRSS2 expression explain organ-specific inhibition of SARS-CoV-2 infection by enzalutamide
Source: Nat Commun. 2021 Feb 8;12:866. doi: 10.1038/s41467-021-21171-x (PMC7870838; doi:10.1038/s41467-021-21171-x)
Supplement: Supplementary file 7 — Reporting Summary [file 41467_2021_21171_MOESM7_ESM.pdf]

nature research

Corresponding author(s): Dong Gao

Last updated by author(s): Jan 15, 2021

Reporting Summary

Nature Research wishes to improve the reproducibility of the work that we publish. This form provides structure for consistency and transparency in reporting. For further information on Nature Research policies, see our Editorial Policies and the Editorial Policy Checklist.

Statistics

For all statistical analyses, confirm that the following items are present in the figure legend, table legend, main text, or Methods section.

n/a	Confirmed	
☐	☒	The exact sample size (*n*) for each experimental group/condition, given as a discrete number and unit of measurement
☐	☒	A statement on whether measurements were taken from distinct samples or whether the same sample was measured repeatedly
☐	☒	The statistical test(s) used AND whether they are one- or two-sided<br>*Only common tests should be described solely by name; describe more complex techniques in the Methods section.*
☐	☒	A description of all covariates tested
☐	☒	A description of any assumptions or corrections, such as tests of normality and adjustment for multiple comparisons
☐	☒	A full description of the statistical parameters including central tendency (e.g. means) or other basic estimates (e.g. regression coefficient) AND variation (e.g. standard deviation) or associated estimates of uncertainty (e.g. confidence intervals)
☐	☒	For null hypothesis testing, the test statistic (e.g. *F*, *t*, *r*) with confidence intervals, effect sizes, degrees of freedom and *P* value noted<br>*Give P values as exact values whenever suitable.*
☒	☐	For Bayesian analysis, information on the choice of priors and Markov chain Monte Carlo settings
☒	☐	For hierarchical and complex designs, identification of the appropriate level for tests and full reporting of outcomes
☐	☒	Estimates of effect sizes (e.g. Cohen's *d*, Pearson's *r*), indicating how they were calculated

Our web collection on statistics for biologists contains articles on many of the points above.

Software and code

Policy information about availability of computer code

Data collection	The methods for our data collection could been seen in methods part that includes “ChIP-seq library preparation”, “ATAC-seq library preparation”
Data analysis	AR ChIP-seq: Raw fastq files were first trimmed to remove adaptors using TrimGalore-0.5.0 with the following parameter settings: -q 25 --phred33 --length 35 -e 0.1 --stringency 4. Trimmed fastq files were then mapped to hg19 genome utilizing Bowtie2-2.3.1. Sambamba_v0.6.6 was conducted to remove duplicates. For IGV-2.3 visualization, deepTools-3.2.1 was then performed using function bamCoverage to generate normalized CPM .bw files. For peak calling, MACS2-2.1.1 was utilized with -q 0.05 parameter setting. DeepTools was further applied for heatmap visualization with the function of computeMatrix and plotHeatmap.<br>ATAC-seq: The approach used or ATAC-seq data processing was quite similar to that used for ChIP-seq data processing. However, the peak calling step differed due to the lack of input control files. In brief, after raw reads were trimmed with TrimGalore-0.5.0, Bowtie2-2.3.1 was used for mapping the reads to the hg19 genome. Samtools-1.4 was further utilized for bam file sorting and indexing. The bamCoverage function in deepTools was used to generate .bw files with counts per million (CPM) normalization. R-3.6.1 package Diffbind-2.12.0 was used to identify overlapped peaks between AR ChIP-seq-generated peaks in LNCaP cells and ATAC-seq-generated peaks in all four cell lines, respectively. Then, both-open peaks were defined by overlapping the above generated peaks in all four cell lines. To further identify specific prostate-open peaks, we employed the intersect function in bedtools-2.25.0 to exclude peaks that emerged in any of the three lung cell lines in LNCaP cells. GraphPad Prism 7 was utilized for statistical analyses.

For manuscripts utilizing custom algorithms or software that are central to the research but not yet described in published literature, software must be made available to editors and reviewers. We strongly encourage code deposition in a community repository (e.g. GitHub). See the Nature Research guidelines for submitting code & software for further information.

Data

Policy information about availability of data

All manuscripts must include a data availability statement. This statement should provide the following information, where applicable:

- Accession codes, unique identifiers, or web links for publicly available datasets
- A list of figures that have associated raw data
- A description of any restrictions on data availability

The raw data and processed data for ChIP-seq and ATAC-seq data are deposited in the Gene Expression Omnibus (GEO) database under GSE163623 (ChIP-seq) and GSE163624 (ATAC-seq) respectively. These data also have been deposited in NODE (http://www.biosino.org/node) under OEX010556 (ChIP-seq) and OEX010557 (ATAC-seq). Public ChIP-seq and RNA-seq datasets used in this study are available from GEO under the following accession code: GSE148277 (ER ChIP-seq in T47D cells), GSE7224949 (ER ChIP-seq in MCF7 cells), GSE148276 and GSE148276 (RNA-seq in T47D and MCF7 cells). Gene expression matrices of multiple cancer cell lines were downloaded from cBioPortal (http://www.cbioportal.org). Source data are provided with this paper.

Field-specific reporting

Please select the one below that is the best fit for your research. If you are not sure, read the appropriate sections before making your selection.

☒ Life sciences ☐ Behavioural & social sciences ☐ Ecological, evolutionary & environmental sciences

For a reference copy of the document with all sections, see nature.com/documents/nr-reporting-summary-flat.pdf

Life sciences study design

All studies must disclose on these points even when the disclosure is negative.

Sample size	The sample size has been stated in the figure legends. This was chosen based on previous experience with similar experiments in the literature. We used minimal numbers of animals, which could still allow for generation of statistically meaningful data. No statistical methods were used to predetermine sample size.
Data exclusions	An Ad-ACE2-transduced WT mouse without SARS-CoV-2 infection was excluded due to a technical issue.
Replication	For in vitro assays, peseudoviral entry evaluation by luciferase, qRT-PCR and western blotting assays were repeated for at least three times. For in vivo assays, comparison the viral loads between Tmprss2-KO and WT mice with SARS-CoV-2 infection were repeated at least twice. In the repeated experiments, similar results were obtained. Use of statistical methods have been described in relevant figure legends. All attempts at replication were successful.
Randomization	Fields with useful information for histology analysis by immunofluorescence and IHC were selected randomly. For experiments involving mice, no randomization of mice was performed. Mice that were used in all experiments were age and sex-matched where possible. For cell line experiments, samples were grouped based on the genotypes.
Blinding	The quantification of the percentage of cells with SARS-CoV-2 infection using IHC staining for S protein was conducted by 4 members of our lab who did not know the samples and antibodies we used. Other experiments does not require investigators to be blinded to group allocation during data collection and/or analysis as they were much more quantitative. Samples were analyzed with the same protocol by different investigators. The data collection was not subjective.

Reporting for specific materials, systems and methods

We require information from authors about some types of materials, experimental systems and methods used in many studies. Here, indicate whether each material, system or method listed is relevant to your study. If you are not sure if a list item applies to your research, read the appropriate section before selecting a response.

Materials & experimental systems

n/a	Involved in the study	
☐	☒	Antibodies
☐	☒	Eukaryotic cell lines
☒	☐	Palaeontology and archaeology
☐	☒	Animals and other organisms
☐	☒	Human research participants
☒	☐	Clinical data
☒	☐	Dual use research of concern

Methods

n/a	Involved in the study	
☐	☒	ChIP-seq
☒	☐	Flow cytometry
☒	☐	MRI-based neuroimaging

Antibodies

Antibodies used	Immunofluorescence and IHC: anti-SPC (Sigma-Aldrich, ab3786, 1:200), anti-AR (Abcam, ab108341, 1:200), and anti-TMPRSS2 (Abcam, ab92323, 1:250), HRP conjugated secondary antibody (OriGene, SP9001);<br>Western blotting: β-Actin (Sigma-Aldrich, A3854, 1:5,000), anti-AR (Abcam, ab108341, 1:2,000), anti-TMPRSS2 (Abcam, ab92323, 1:1,000), and anti-ACE2 (Proteintech, 21115-1-AP, 1:1000), anti-ER alpha(Santa Cruz, sc-543,1:1,000)
Validation	Validation of each antibody was conducted by manufactures and validation statements and references for these antibodies can be found on the manufacture's websites.<br>Anti-AR antibody: Species Reactivity: mouse, human. Usage: Western blot, IF, IHC<br>Anti-TMPRSS2: Species Reactivity: mouse, human. Usage: Western blot, IF, IHC<br>Anti-ACE2: Species Reactivity:mouse, human. Usage: Western blot<br>Anti-FLAG: Species Reactivity: mouse, human. Usage: IHC<br>Anti-SPC: Species Reactivity: mouse, human. Usage: IF<br>Anti-ER alpha: Species Reactivity: mouse. Usage: Western blot

Eukaryotic cell lines

Policy information about cell lines

Cell line source(s)	Cell lines H2126, H1437, A549 and Calu3 were purchased from the Cell Bank of the Chinese Academy of Sciences (Shanghai, China). LNCaP, VCaP, 22RV1, DU145 (Supplementary Figure 2b), LAPC4 (Supplementary Figure 2b), PC3 (Supplementary Figure 2b), HEK293F, 293T were purchased from ATCC. MSKPCa1 and MSKPCa3 were previously established by our lab.
Authentication	All cell lines were kept at low passages to maintain their identity. The mRNA and protein expression of identity-related genes was confirmed with qRT-PCR and western blot respectively.
Mycoplasma contamination	All the cell lines were negative for mycoplasma contamination
Commonly misidentified lines<br>(See ICLAC register)	None of the lines used are included in ICLAC register

Animals and other organisms

Policy information about studies involving animals; ARRIVE guidelines recommended for reporting animal research

Laboratory animals	TMPRSS2-KO mouse line was previously constructed by our lab. All GEMMs were with C57BL/6 background. 10-18 week old mice were used. Both male and female mice were used for the study.
Wild animals	Wild animals were not included in our study
Field-collected samples	Field-collected samples were not included in our study
Ethics oversight	All animal experiments were performed under protocols approved by the Institutional Animal Care and Use Committee of Shanghai Institute of Biochemistry and Cell Biology.

Note that full information on the approval of the study protocol must also be provided in the manuscript.

Human research participants

Policy information about studies involving human research participants

Population characteristics	Human lung samples were adjacent normal tissue obtained from patients who suffering from lung cancer aged from 42 to 71 and underwent pulmonary lobectomy in 2020.
Recruitment	Samples with pathological examination which were confirmed to be the adjacent normal tissue in lung cancer patients were collected.
Ethics oversight	All clinical samples were anonymously coded, and the protocol was approved by the institutional review board of CEMCS.

Note that full information on the approval of the study protocol must also be provided in the manuscript.

ChIP-seq

Data deposition

- [x] Confirm that both raw and final processed data have been deposited in a public database such as GEO.
- [x] Confirm that you have deposited or provided access to graph files (e.g. BED files) for the called peaks.

Data access links<br>*May remain private before publication.*	The raw data and processed data for ChIP-seq are deposited in the Gene Expression Omnibus (GEO) database under GSE163623 (ChIP-seq).These data also have been deposited in NODE (http://www.biosino.org/node) under OEX010556 (ChIP-seq).
Files in database submission	Raw data and analysis data of AR ChIP-seq (A549, H1437, H2126 and LNCaP).
Genome browser session<br>(e.g. UCSC)	All data could be visualized by IGV viewer using our accessible bigwig files.

Methodology

Replicates	AR ChIP-seq were performed on 4 samples: A549, H1437, H2126 and LNCaP with no replicates. Also, there was one matched “input-mix” sample, which has been cross-linked and sonicated but not immuno-precipitated
Sequencing depth	For the AR ChIP-seq experiments, total number of reads of Input-mix, A549, H1437, H2126 and LNCaP are 32,351,108, 43,972,542, 37,304,216, 48,024,793, 33,433,167. All reads are paired-end with 150 bp
Antibodies	3 µL of anti-AR antibody (Abcam, ab108341)
Peak calling parameters	macs2 callpeak -c -t -q 0.05 -f BAM -g hs -n --outdir
Data quality	Raw fastq files were first trimmed to remove adaptors using TrimGalore-0.5.0 with the following parameter settings: -q 25 --phred33 --length 35 -e 0.1 --stringency 4. Trimmed fastq files were then mapped to hg19 genome utilizing Bowtie2-2.3.1. Sambamba_v0.6.6 was conducted to remove duplicates.
Software	The following software and tools for ChIP-seq were performed, including Bowtie2 to map reads to reference genome, samtools-1.4 to filter low-quality reads and duplicates, MACS2-2.1.1 to call peaks and deeptools-3.2.1 to visualize the data as heatmap and profile plots
