# Supplementary material for: Distinct mechanisms for TMPRSS2 expression explain organ-specific inhibition of SARS-CoV-2 infection by enzalutamide
Source: Nat Commun. 2021 Feb 8;12:866. doi: 10.1038/s41467-021-21171-x (PMC7870838; doi:10.1038/s41467-021-21171-x)

Supplemental figure 2b

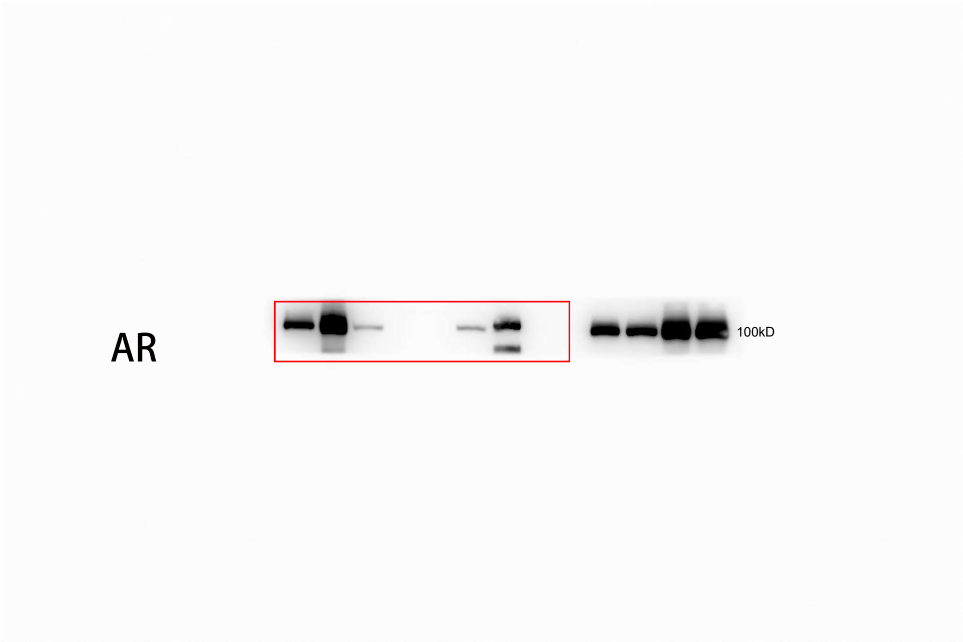

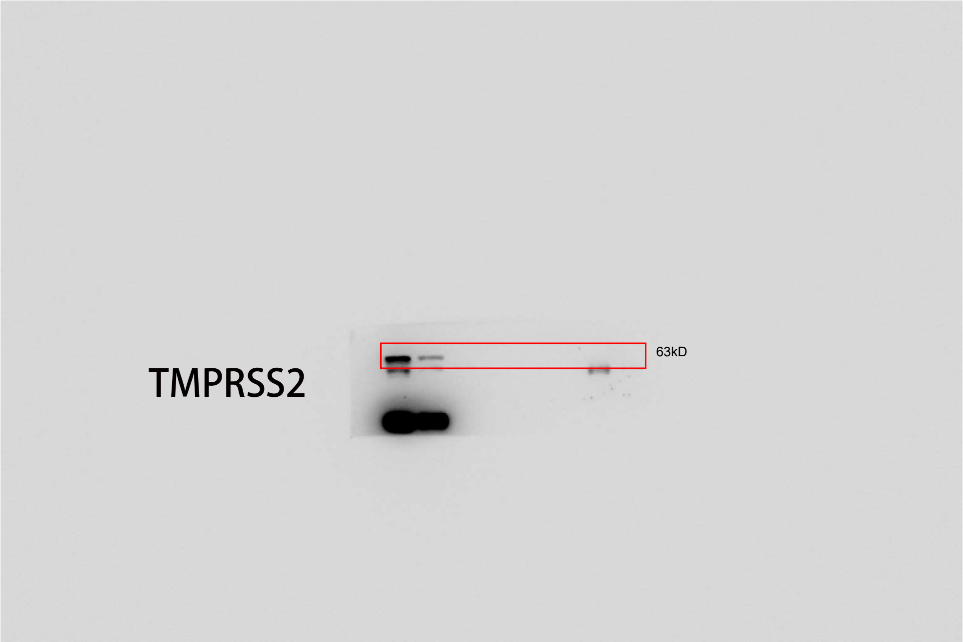

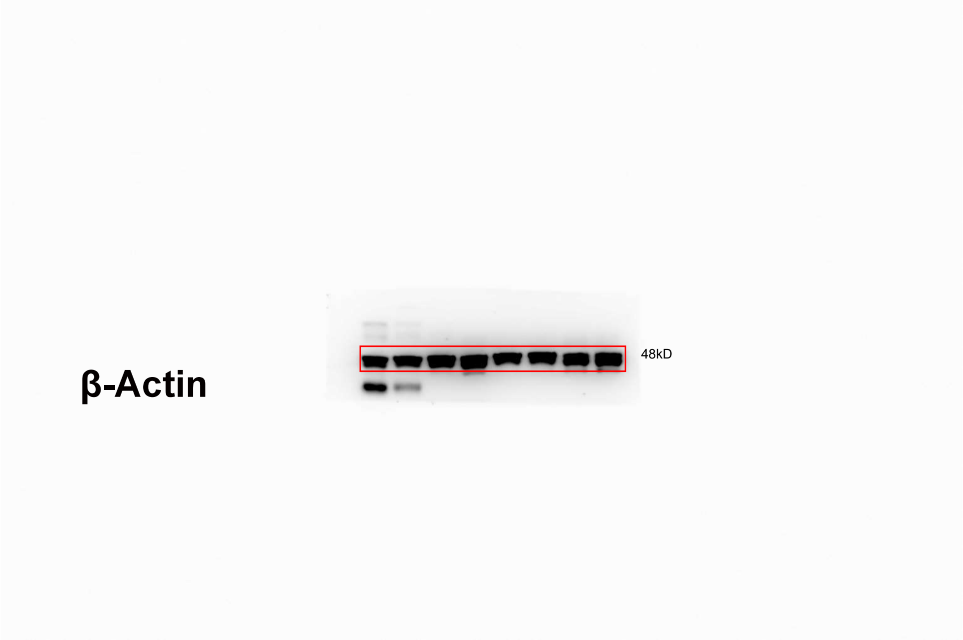

Supplemental figure 2c and 2d

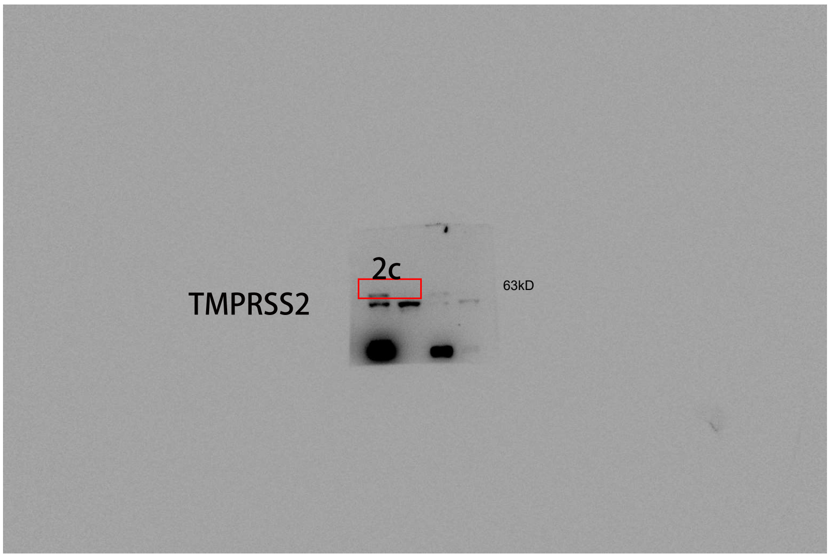

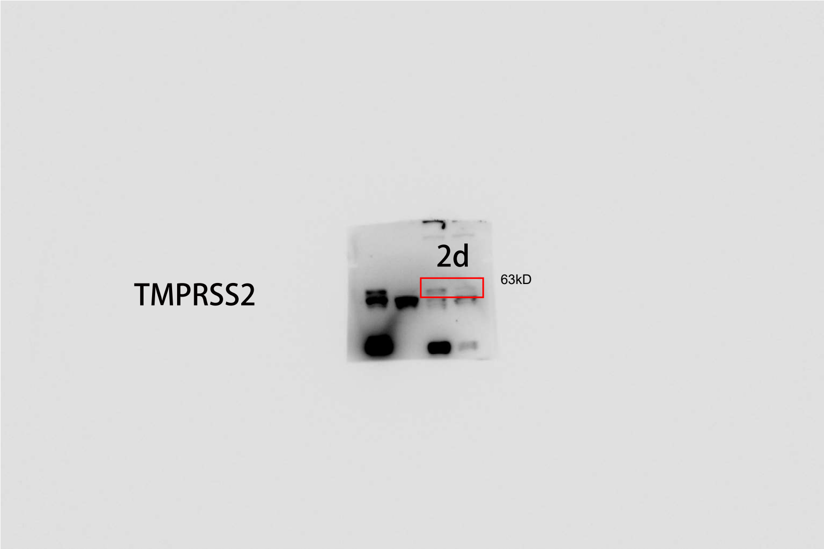

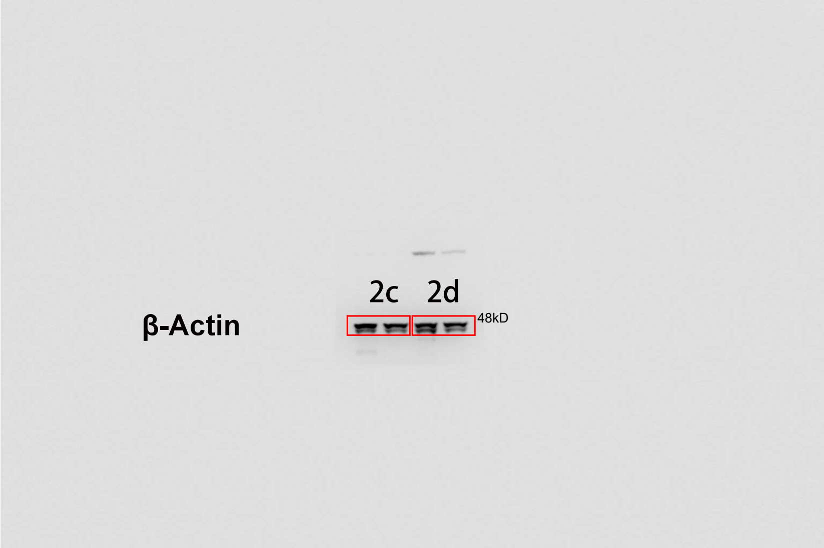

Supplemental figure 3a and 3c

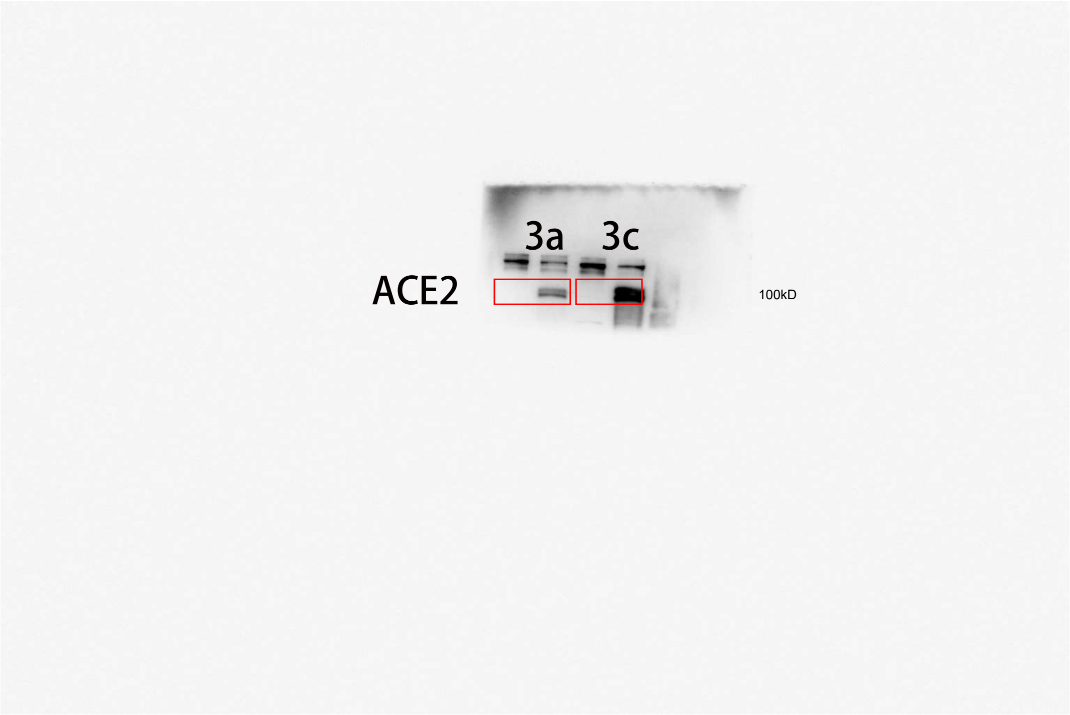

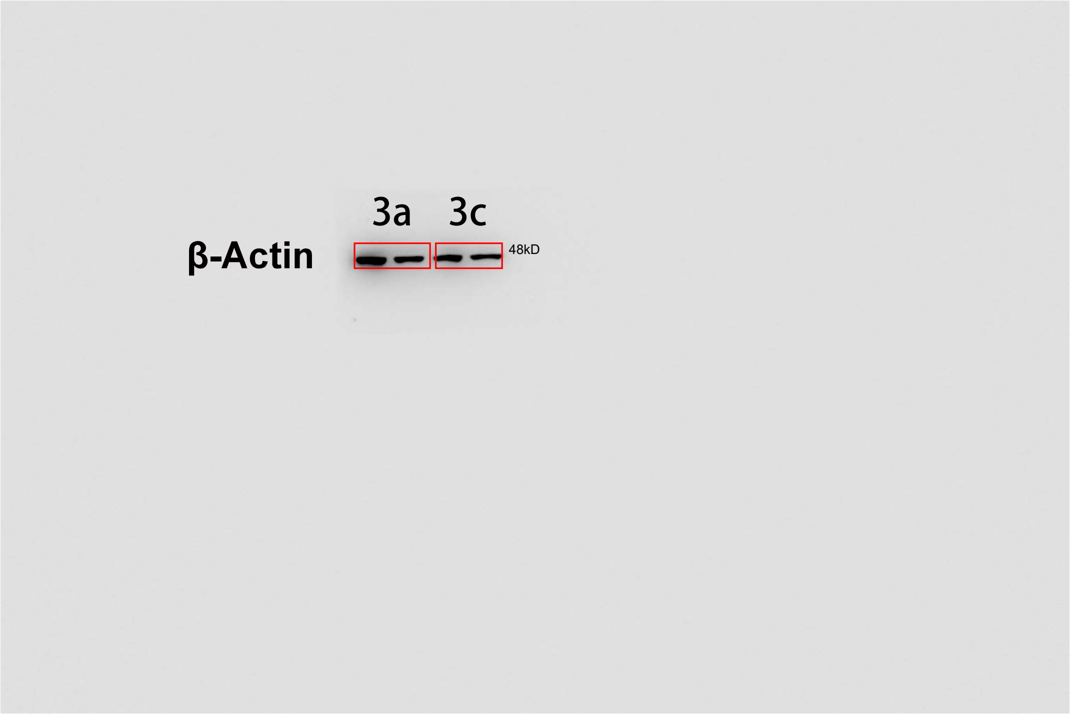

Supplemental figure 5a

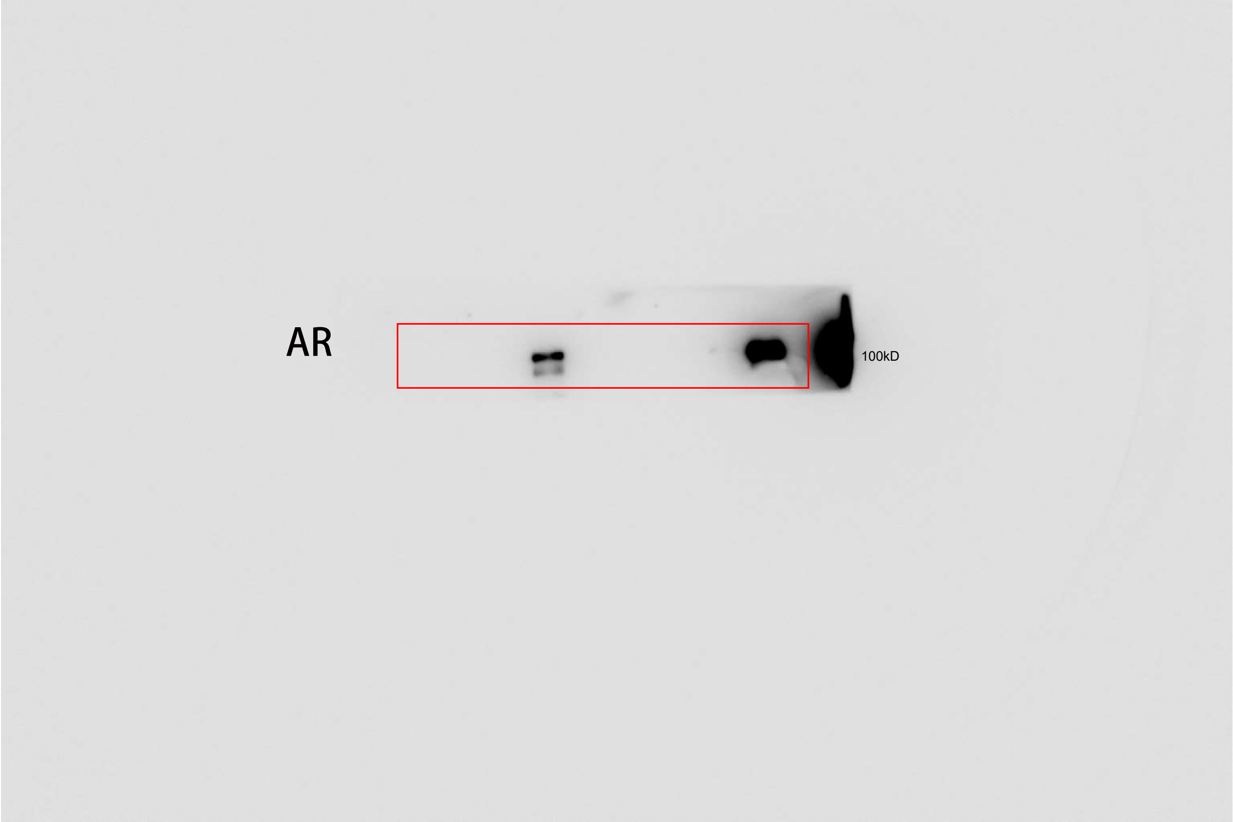

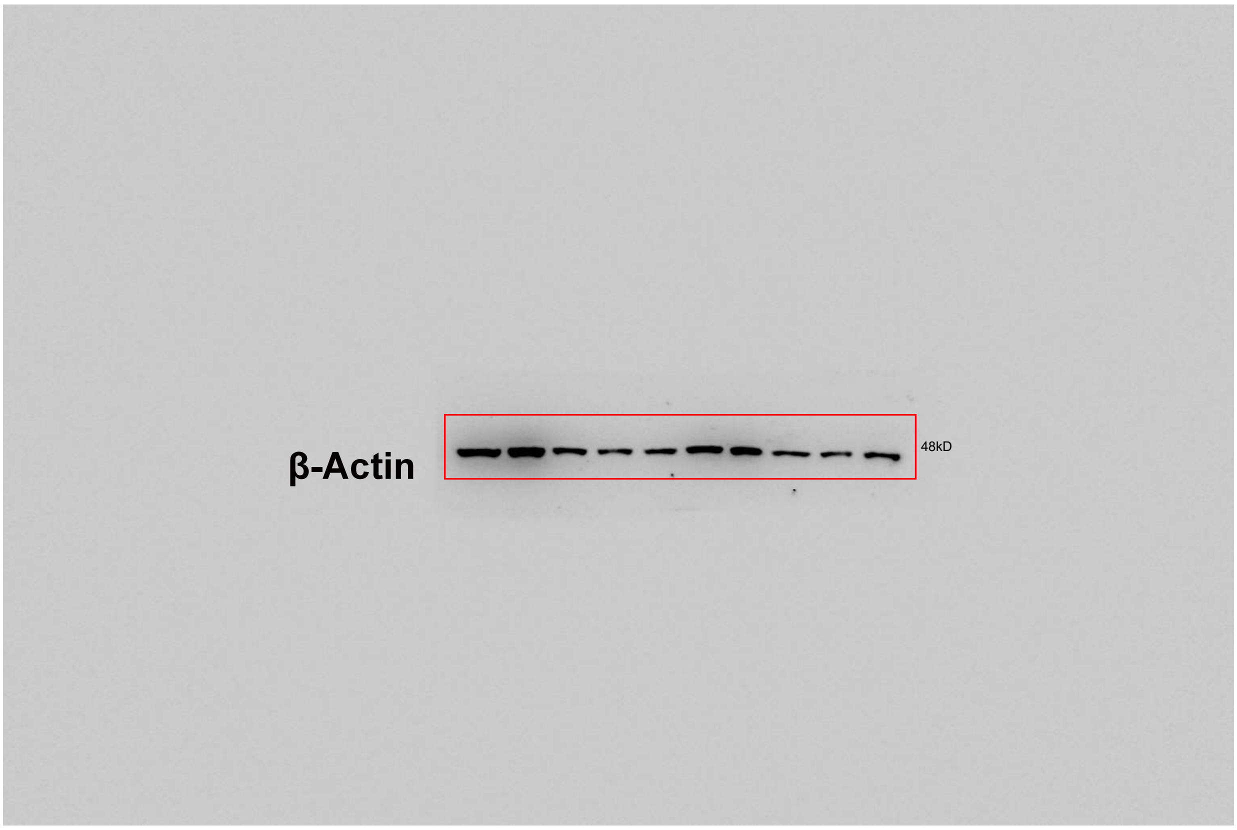

Supplemental figure 5h

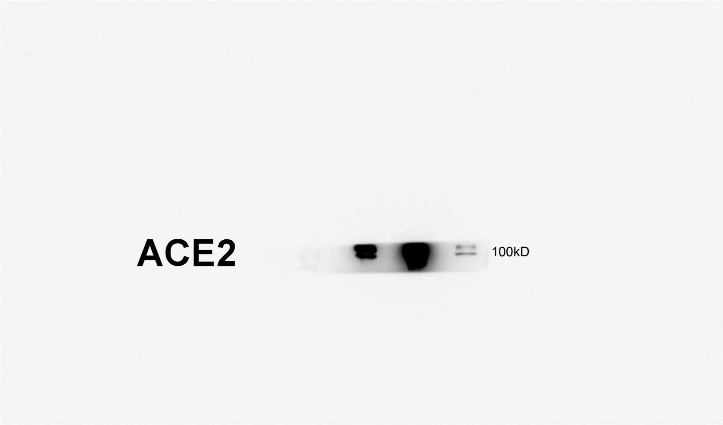

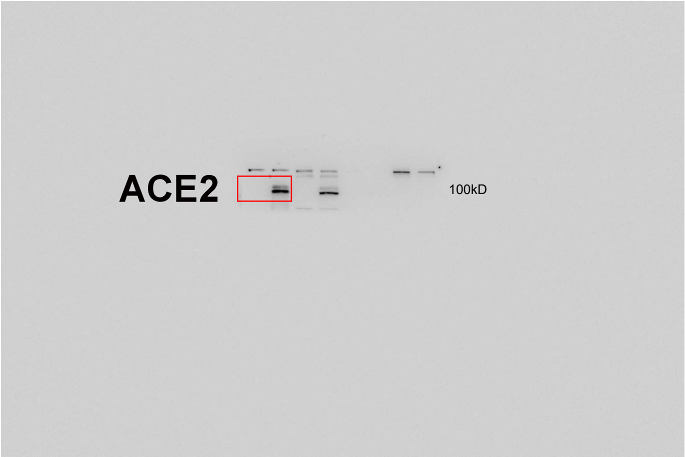

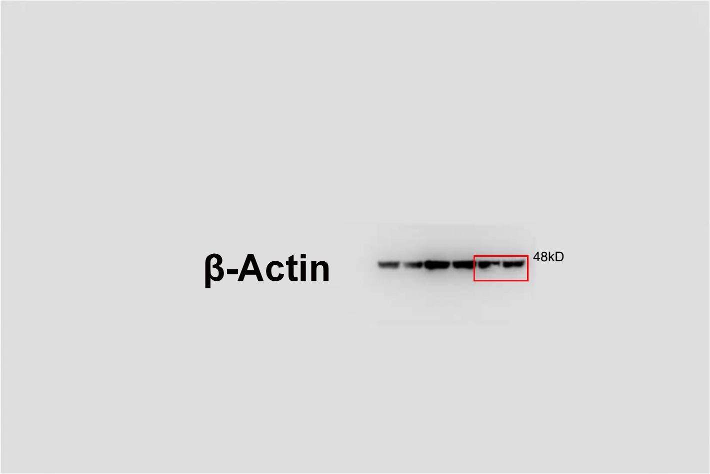

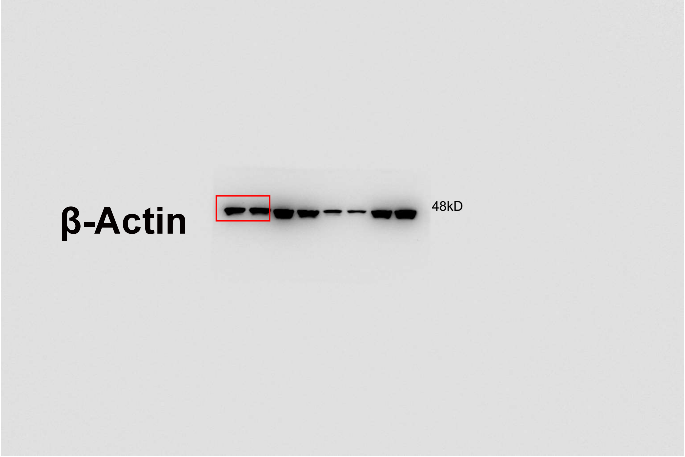

Supplemental figure 8c

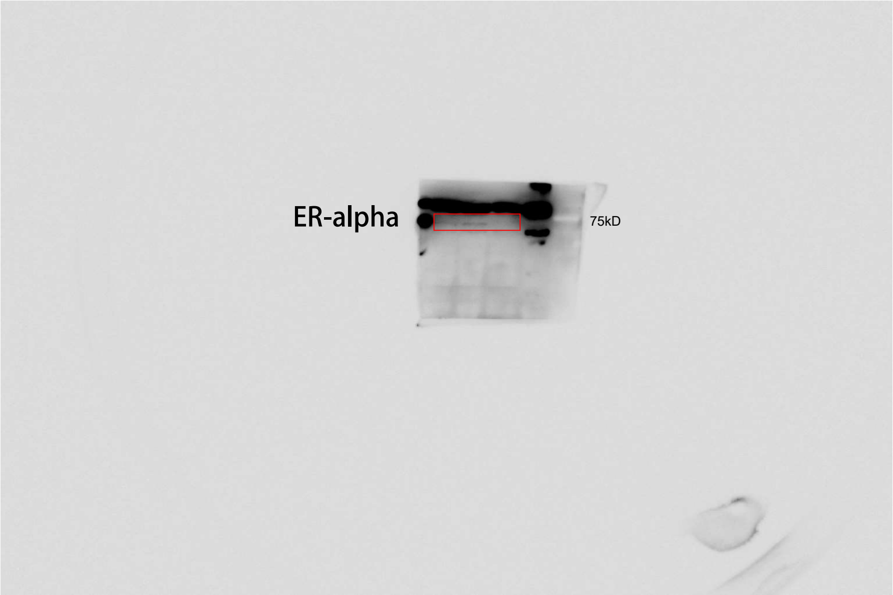

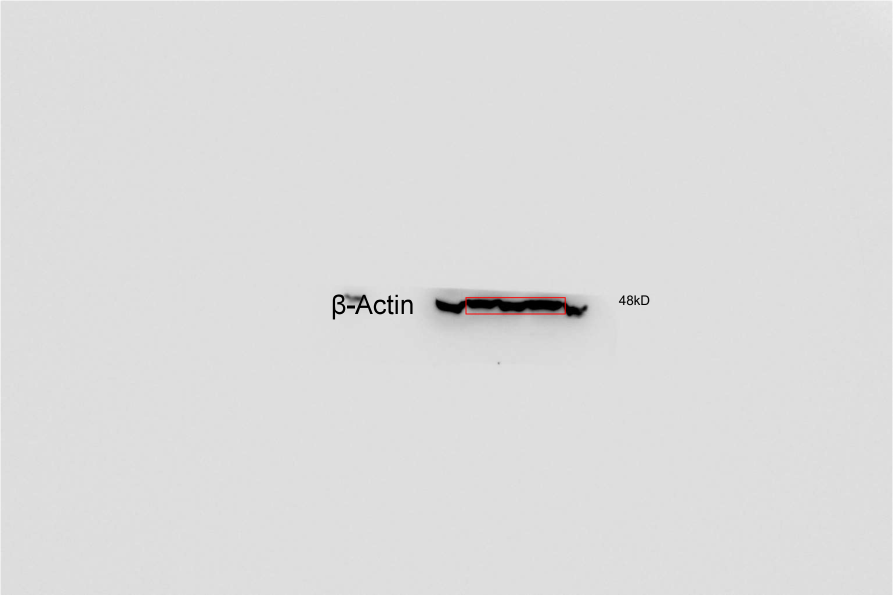

Supplement: Supplementary file 8 — Source Data [file 41467_2021_21171_MOESM8_ESM.pdf]
